# Metal-Based Nanomaterials for the Sensing of NSAIDS

**DOI:** 10.3390/nano14070630

**Published:** 2024-04-04

**Authors:** Farah Quddus, Afzal Shah, Naimat Ullah, Iltaf Shah

**Affiliations:** 1 Department of Chemistry, Quaid-I-Azam University, Islamabad 45320, Pakistan; farahquddus931@gmail.com (F.Q.); naimatullah@chem.qau.edu.pk (N.U.); 2 Department of Chemistry, College of Science, United Arab Emirates University, Al Ain P.O. Box 15551, United Arab Emirates

**Keywords:** cadmium sulfide, zinc oxide, NSAIDs, naproxen, mobic

## Abstract

Cadmium sulfide and zinc oxide nanoparticles were prepared, characterized and used as electrode modifiers for the sensing of two non-steroidal anti-inflammatory drugs (NSAIDs): naproxen and mobic. The structural and morphological characterization of the synthesized nanoparticles was carried out by XRD, UV-Vis spectroscopy, FTIR and scanning electron microscopy. The electrode’s enhanced surface area facilitated the signal amplification of the selected NSAIDs. The CdS-modified glassy carbon electrode (GCE) enhanced the electro-oxidation signals of naproxen to four times that of the bare GCE, while the ZnO-modified GCE led to a two-fold enhancement in the electro-oxidation signals of mobic. The oxidation of both NSAIDs occurred in a pH-dependent manner, suggesting the involvement of protons in their electron transfer reactions. The experimental conditions for the sensing of naproxen and mobic were optimized and, under optimized conditions, the modified electrode surface demonstrated the qualities of sensitivity and selectivity, and a fast responsiveness to the target NSAIDs.

## 1. Introduction

Electroanalytical techniques are pivotal in several critical domains such as medicine, clinical analysis, pharmaceutical research and environmental remediation. Within this realm, voltammetric methods utilizing modified electrodes based on nanomaterials have emerged as a focal point. Electrochemical techniques have gained widespread recognition due to their exceptional sensitivity and selectivity, enabling both a qualitative and quantitative assessment of their target analytes, especially electroactive drugs. Of the electroactive drugs, NSAIDs are most frequently used. Hence, a sensitive platform for their analysis is mandatory for proving the validity of label claims and garnering insights about the action mechanism of NSAIDs.

NSAIDs are commonly prescribed for the treatment of pain and inflammation [1]. They possess antipyretic and analgesic characteristics [2]. Approximately 5–10% of the medicines prescribed annually are NSAIDs [3]. Some common examples of NSAIDs are ibuprofen, naproxen, piroxicam, mobic, flurbiprofen, etc. Naproxen and mobic are widely used NSAIDs for the treatment of a number of diseases such as fever, inflammation, pain, osteoarthritis and rheumatoid arthritis [4,5]. These are broad-spectrum anti-inflammatory drugs used due to their potential for treating moderate to severe pain. Due to the medicinal effectiveness and redox properties of NSAIDs, the current work is focused on an electroanalysis of naproxen and mobic.

Due to widespread usage of NSAIDs, a significant amount of their residual products enter into the environment without any pretreatment or proper disposal systems. Their improper disposal by pharmaceutical industries and hospitals may result in water contamination, leading to adverse effects on flora and fauna. Therefore, a sensitive analytical tool is necessary for the early detection of water contaminants. In this regard, electrochemical sensors are a promising choice [4].

Nanomaterial-based sensing platforms are currently the leading choice for drug detection and analysis [6]. The advancement in nanotechnology has led to a diverse range in the applications of nanoparticles in medicine, pharmaceutical companies, electronic devices, electrochemical sensors, drug delivery and environmental remediation. Cadmium sulfide (CdS) nanoparticles are used in solar cells, batteries and electronics appliances due to their catalytic, optical and electrical properties [7,8]. The electrical and optical properties of nanoparticles can be tuned to some extent by adjusting their reaction conditions to obtain a size, shape and morphology of the nanomaterials closer to the desired choice [9].

**Scheme 1:**
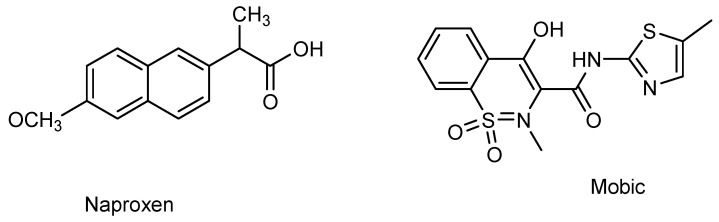
Chemical structures of naproxen and mobic.

Semiconductor oxide nanoparticles are attracting the attention of researchers due to their effectiveness in electrocatalysis and photocatalysis [10]. Of the various semiconductor materials, CdS is appealing choice due to its preferred band gap, and ZnO is extensively used due to its high biocompatibility and environmentally benign characteristics [11,12]. Both CdS and ZnO are n-type semiconductors. They have been reported as efficient electrocatalysts and photocatalysts for the detection and photocatalytic breakdown of pharmaceutical drugs [13,14]. Hence, the current work presents CdS and ZnO as electrocatalysts and photocatalysts for the electroanalysis of the NSAIDs naproxen and mobic. The purpose of the current work is to develop a sensitive electrochemical platform for the detection of two representative NSAIDs (i.e., mobic and naproxen). Scheme 1 demonstrates the chemical structures of mobic and naproxen.

## 2. Experimental Section

### 2.1. Materials and Instrumentation

Naproxen and mobic were obtained from a pharmaceutical company in Peshawar, Pakistan. Cadmium acetate dihydrate Cd(CH_3_COO)_2_·2H_2_O and thiourea (NH_2_CSNH_2_) were obtained from Sigma Aldrich and used as precursors for the synthesis of CdS nanoparticles. Zn(NO_3_)_2_·6H_2_O, for the preparation of ZnO nanoparticles, was obtained from Sigma Aldrich. NaOH, KCl, HCl, Britton–Robinson buffer (BRB) and phosphate-buffered saline (PBS) were examined as supporting electrolytes. PBS was prepared by dissolving a specified amount of Na_2_HPO_4_ and NaH_2_PO_4_ in distilled water and using 0.1 M HCl and 0.1 M NaOH for pH adjustment. Supporting electrolytes in the pH range of 2–10 were prepared using PBS buffer solutions. Electrochemical measurements were carried out using Metrohm Auto lab and NOVA 1.11 software. Gamry Software (Gamry Interface 5000E Potentiosta equipped with software 7.9.0) was used for the investigation of electrochemical impedance spectroscopy (EIS). For pH measurements, INOLAB pH meter model 720 was used. Micro volume measurements were carried out using EP-10 and EP-100 as well as motorized µL pipettes.

### 2.2. Synthesis of Nanoparticles

A calculated amount of cadmium nitrate tetrahydrate (1 M) and thiourea (1 M) were dissolved in 50 mL of distilled water, followed by constant magnetic stirring for 1 h. During this process, argon gas was passed through the resulting mixture to bubble out all the trapped oxygen from the reaction mixture. Afterwards, the reaction solution was ultrasonicated for 2 h at 80 °C. Orange- to yellowish-colored precipitates were formed, followed by their filtering and washing with distilled water and ethanol, and finally dried in an oven at 60 °C. ZnO nanoparticles were prepared according to the literature-reported method [15].

### 2.3. Modification of the Glassy Carbon Electrode (GCE)

Prior to analysis, the GCE was cleaned to a smooth and shiny surface. The cleaning involved a nylon rubbing mat and an aqueous alumina slurry of 1 µm particle size. The GCE was gently rubbed on the mat in a figure-eight pattern, resulting in a polished and smooth surface. However, to ensure further cleaning, 10 min sonication in a solution composed of ethanol, acetone and water, followed by air drying, was carried out. The drop casting method was employed for the preparation of a modified sensing platform for the detection of naproxen and mobic. For this purpose, a 1.5 mg/mL slurry of cadmium sulfide nanoparticles was prepared in DMF solvent, followed by sonication. For the fabrication of the glassy carbon electrode’s surface, 10 µL of the prepared slurry was poured onto it, followed by drying for 3–4 min. After the adsorption of the slurry, a naproxen solution was also poured, using the drop casting method, onto the surface of the modified glassy carbon electrode. Finally, the electrode modified with naproxen was dipped into an electrolyte solution to record its voltammograms. The same procedure was adopted for mobic using a zinc oxide nanoparticle-modified electrode. A three-electrode assembly consisting of Ag/AgCl as the reference electrode, platinum wire as the counter electrode and the glassy carbon electrode (GCE) as the transducer of the working electrode was used for electrochemical studies.

## 3. Results and Discussion

### 3.1. Structural and Morphological Characterization

**Figure 1:**
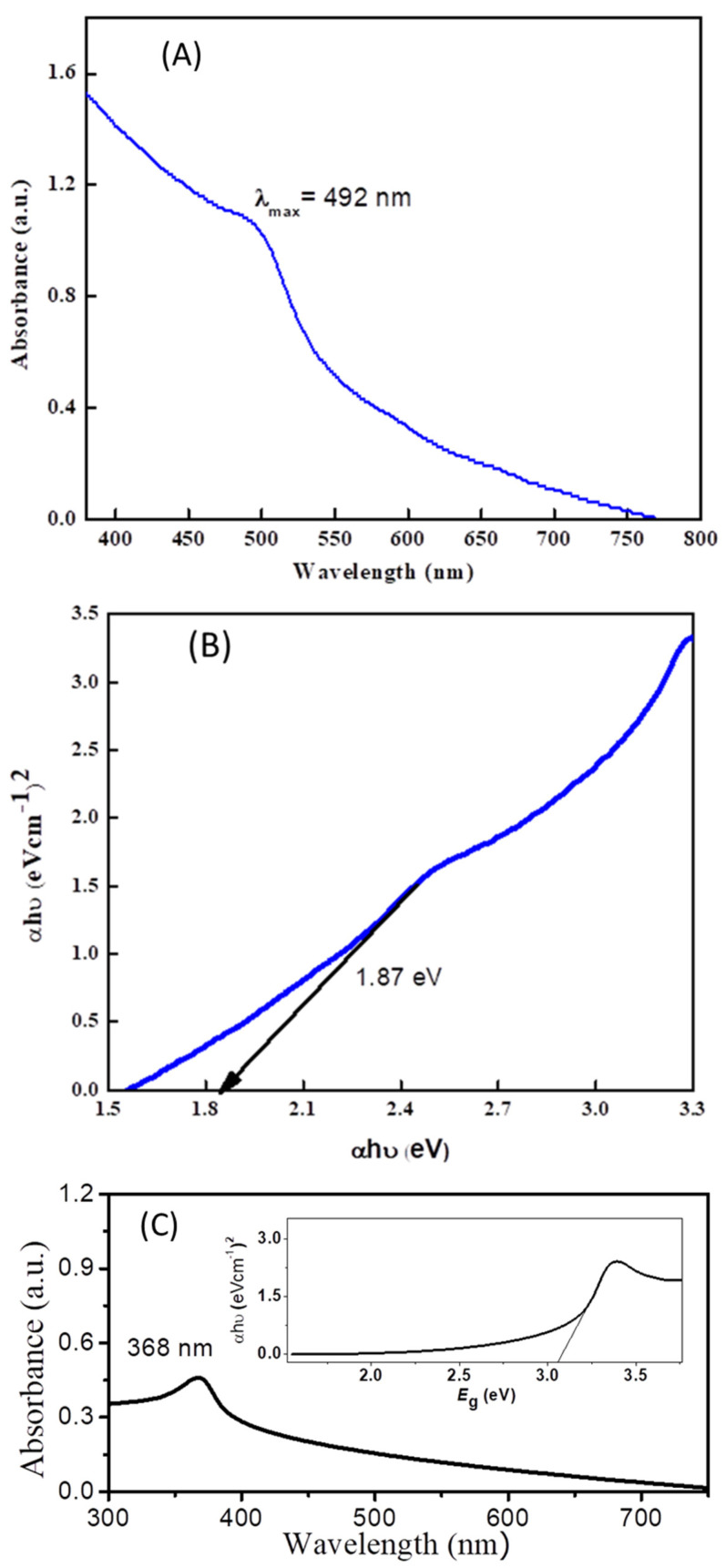
(**A**) UV-VIS spectrum of cadmium sulfide nanoparticles. (**B**) Tauc plot showing band gap of cadmium sulfide nanoparticles. (**C**) UV-VIS spectrum of ZnO with inset Tauc plot for the estimation of the band gap of ZnO.

The optical and electronic properties of cadmium sulfide and zinc oxide nanoparticles were studied via UV-VIS spectroscopy. An extended absorption of CdS in the range of 300–490 nm, with the maximum absorption occurring at 492 nm, is obvious from Figure 1A. This broad absorption signal is observed due to quantum confinement and size quantization. A band gap with a value of 1.87 eV was calculated by constructing a Tauc plot (Figure 1B) using the data obtained from the UV-Vis absorption spectrum. Similarly, the band gap (3.1 eV) of ZnO was calculated from the Tauc plot of its UV-Vis absorption spectrum (Figure 1C). 

The band gap was evaluated according to the following equation [16]:

$$\alpha \;h\nu =A(h\nu -Eg{)}^{n}$$

**Figure 2:**
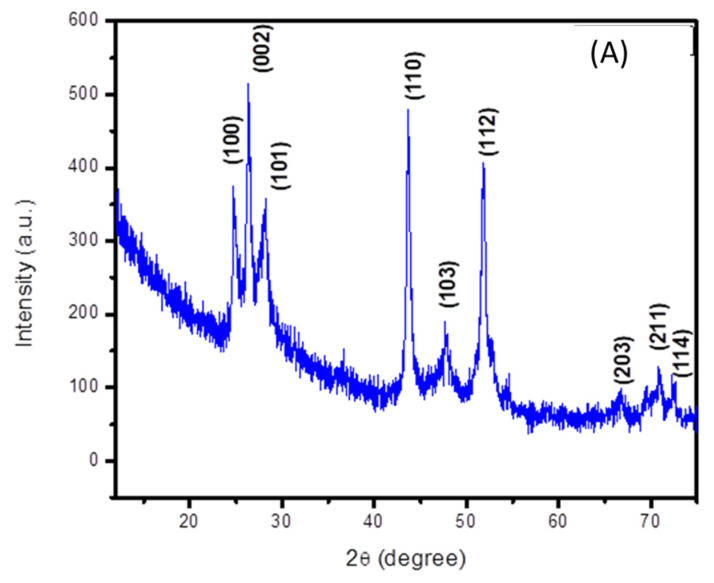

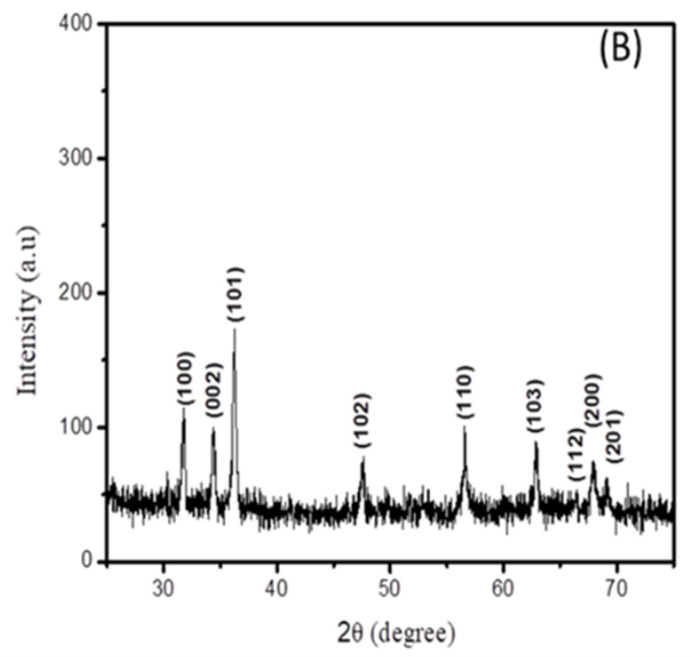
XRD spectra of (**A**) cadmium sulfide and (**B**) zinc oxide nanoparticles.

The crystalline structure of cadmium sulfide was analyzed using its X-ray diffraction spectrum. Its peak positions match with the standard JCPDS card no. 06-0314, which confirms the formation of a hexagonal structure of CdS [17]. The corresponding peaks are indexed in the (100), (002), (101), (103), (112), (203), (211) and (114) planes, as depicted in Figure 2A. Well-defined and distinct peaks indicate the crystallinity of the prepared cadmium sulfide. An average crystallite size of 11 nm was calculated by applying the Debye Scherrer equation.

(1) D=Kλ/βCosθ

The XRD spectrum of the zinc oxide nanoparticles is displayed in Figure 2B. It can be seen that all peak positions are in agreement with the standard JCPDS no 36-1451 [18], indicating the formation of a hexagonal wurtzite structure of ZnO.

**Figure 3:**
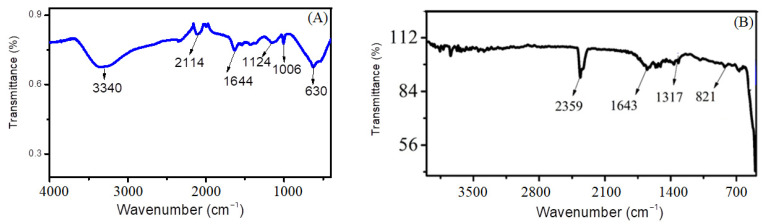
FTIR graph of (**A**) cadmium sulfide nanoparticles and (**B**) zinc oxide nanoparticles.

The presence of functional groups and organic functionalities is confirmed by stretching and bending vibrations, by establishing the Fourier-transform infrared spectrum of the synthesized nanomaterial. Multiple peaks are observed, indicating specific sorts of vibrations; for example, the broad peak found in the region of 3340 cm^−1^ indicates the stretching vibration of the O-H bond present in the water molecule used as a solvent. The peak found at 630 cm^−1^ indicates a stretching vibration, confirming the formation of cadmium sulfide [19]. The stretching bands present at 1006 cm^−1^ and 1124 cm^−1^ indicate the vibration of C-N and C=S due to the use of thiourea as a precursor material. The characteristic peaks present at 1644 cm^−1^ and 2114 cm^−1^ represent C-O stretching and N=C vibrations, respectively [16,20]. Figure 3A represents the FTIR graph for CdS.

Figure 3B shows FTIR spectrum of ZnO nanoparticles. The characteristic Zn-OH band is located at 821 cm^−1^ and the C-O band at 1317 cm^−1^. The peak at 524 cm^−1^ depicts the bending vibration of the Zn-O bond [21]. The presence of these peaks confirmed the chemical bonding of zinc and oxygen (Zn-O). The peak present at 1643 cm^−1^ confirms the presence of some organic functionalities or aromaticity present due to the use of lemon peel extract [22]. The presence of a distinct peak at 2359 cm^−1^ corresponds to the asymmetric stretching vibration of C=N=O. Other certain peaks are also seen in the FTIR spectra that can be attributed to the phytochemicals present in the lemon extract, such as phenol groups, alkaloids, proteins and flavonoids, contributing to the stabilization of the nanoparticles. These biomolecules act as the reducing/stabilizing agents for the formation of ZnO nanoparticles [23].

**Figure 4:**
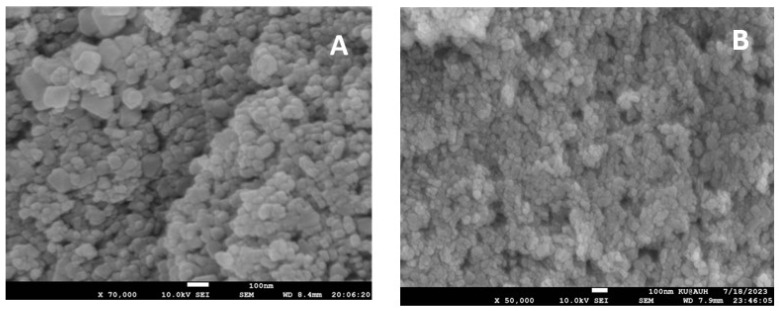
SEM micrographs of (**A**) CdS and (**B**) ZnO nanoparticles.

The surface morphology of these synthesized nanoparticles was analyzed by performing a scanning electron microscopic analysis, as illustrated in Figure 4A. A mostly uniform array of spherical or nearly spherical particles with clumped and aggregated patterns can be seen in the SEM micrograph. The particles were found to be monodisperse and in some places polydisperse particles are seen. The nanoparticles overlapped with each other to form some clusters due to their high surface energy. A SEM image of ZnO is displayed in Figure 4B, from a microscope operated at 10 kV, and images were captured at a magnification of 100 nm. In the SEM image, ZnO is depicted as small spherical-shaped particles with little agglomeration. The size distribution of the particles was determined by nano measuring software. It is inferred from these results that, in the case of ZnO, the particle size ranges from 21 nm to 73 nm, while, for CdS, the size ranges from 19 nm to 119 nm.

### 3.2. Electrocatalytic Role of the Synthesized Nanoparticles in NSAIDs Analysis

**Figure 5:**
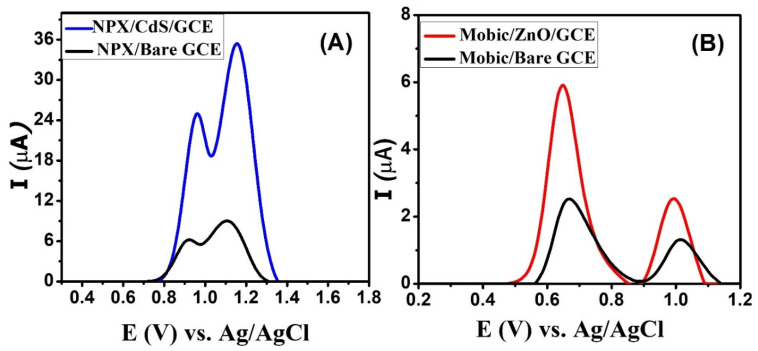
SWVs of 50 µM (**A**) naproxen obtained from bare and CdS-modified electrodes in PBS solution of pH 7 and (**B**) mobic from bare and zinc oxide-modified electrodes in BRB solution of pH 4.

Square-wave voltammograms (SWV) of naproxen were scanned using bare and modified glassy carbon electrodes. In comparison to the signal of naproxen from the bare GCE, a significant increase in the signal’s intensity was noticed due to the catalytic role of CdS on the modified GCE, as depicted in the voltammograms depicted in Figure 5A. Two oxidation peaks are observed at 0.96 and 1.15 V. Thus, CdS nanoparticles enhance the sensitivity of glassy carbon electrodes for the detection of naproxen. Figure 5B shows the role of zinc oxide nanoparticles in enhancing the signal intensity of mobic. Its peaks are slightly shifted towards the low-oxidation-potential region, indicating the better electrocatalytic behavior of the electrocatalysts immobilized over the GCE surface. These sensors lead to the intensification of the signals of NSAIDs by facilitating efficient charge transduction via the bridging role of the electrode modifier between the host (transducer) and the guest (NSAID).

**Figure 6:**
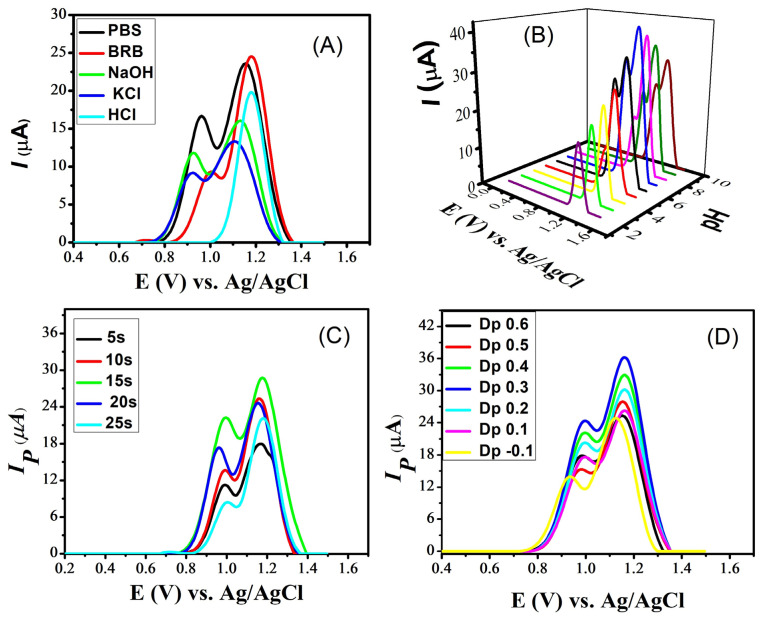
SWVs obtained at a scan rate of 100 mVs^−1^ on cadmium sulfide-modified glassy carbon electrode, showing the effect of (**A**) supporting electrolytes on the signals of naproxen; (**B**) pH of the PBS solutions, in the pH range 2–10, on the signals of naproxen; (**C**) deposition time on the peak current response of naproxen in a PBS solution of pH 7; and (**D**) deposition potential on the peak current of naproxen in a PBS solution of pH 7.

For the further enhancement of the naproxen signals, various experimental conditions were optimized. These include the supporting electrolyte, the optimization of the pH of the medium, the accumulation potential and deposition time. The selection of a suitable supporting electrolyte is crucial for the electrochemical sensing of analytes. Different supporting electrolytes create different responses in the sensing of a particular analyte because the peak shape and peak current of analytes are significantly affected by electrolytes and the pH of the medium. For this purpose, square-wave voltammograms of naproxen from the GCE modified with CdS were scanned in multiple supporting electrolytes such as phosphate-buffered saline (PBS), Britton–Robinson Buffer (BRB), sodium hydroxide, potassium chloride and hydrochloric acid. Intense signals of naproxen were noticed in PBS and BRB compared to the other electrolytes (Figure 6A). Of these, PBS was selected due to the appealing intensity of both peaks of naproxen seen with it. Hence, PbS was selected for further electrochemical experiments on naproxen. After selecting the most suitable electrolyte, its pH was optimized by preparing solutions of PBS in the pH range 2–10. Looking at Figure 6B reveals that PBS of pH 7 is a more suitable medium for naproxen’s electroanalysis because its voltammogram shows distinct peaks with maximum peak currents. Moreover, two peaks of naproxen appear in basic and neutral media, while the first peak does not appear in acidic conditions.

Optimizing deposition potential and deposition time are essential for improving the sensitivity of a sensing platform. The deposition time influences the amount of analyte that accumulates on the sensor’s surface. Longer deposition times generally result in higher peak currents because more analyte molecules have time to accumulate and participate in the electrochemical reaction. For this purpose, the deposition time was varied from 5 s to 25 s and the maximum response was shown at 15 s, as illustrated in Figure 6C. The deposition potential determines the potential at which the analyte is electrochemically deposited or reduced/oxidized on the working electrode. Changing the deposition potential leads to a shift in the peak current. Different analytes may have different electrochemical behaviors at various deposition potentials. By optimizing the deposition potential, an enhancement in the selectivity of a sensor for a specific analyte is achieved. For the selected analyte (naproxen), the deposition potential varied from −1 to +0.6 V and a maximum value to the peak current was obtained at 0.3 V, as shown in Figure 6D.

Similarly, the influence of the supporting electrolytes, pH of the medium, deposition time and potential was examined on the electro-oxidation response of mobic. The results revealed that BRB of pH 4 is the best medium for generating the oxidative response of mobic.

**Scheme 2:**
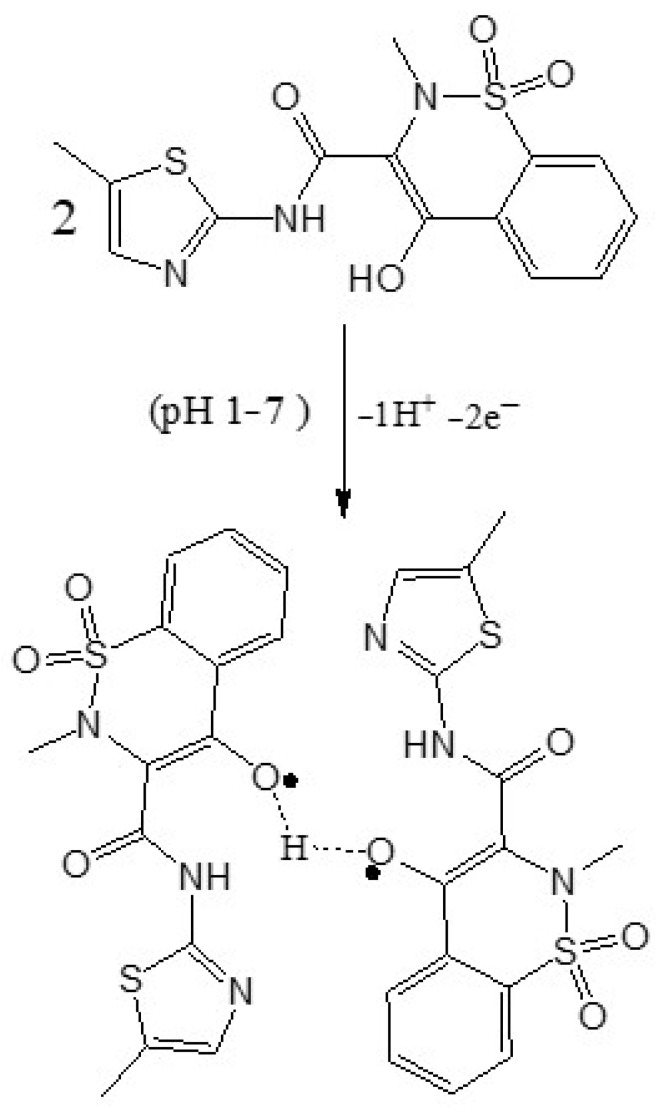
Proposed oxidation mechanism of mobic.

**Scheme 3:**
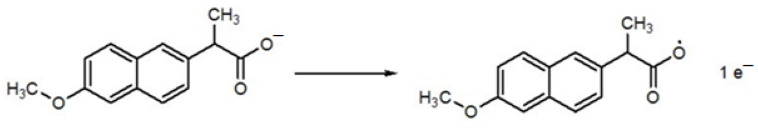
Proposed oxidation mechanism of naproxen.

Scheme 2 shows the proposed mechanism of mobic, which involves proton-coupled electron transfer during oxidation. Scheme 3 represents the proposed electro-oxidation of naproxen. The products of both mobic and naproxen are suggested to adsorb to the sensor surface because the signals of both NSAIDs decreased in intensity with consecutive scanning without cleaning the electrode in between the consecutive scans.

### 3.3. Analytical Detection

**Figure 7:**
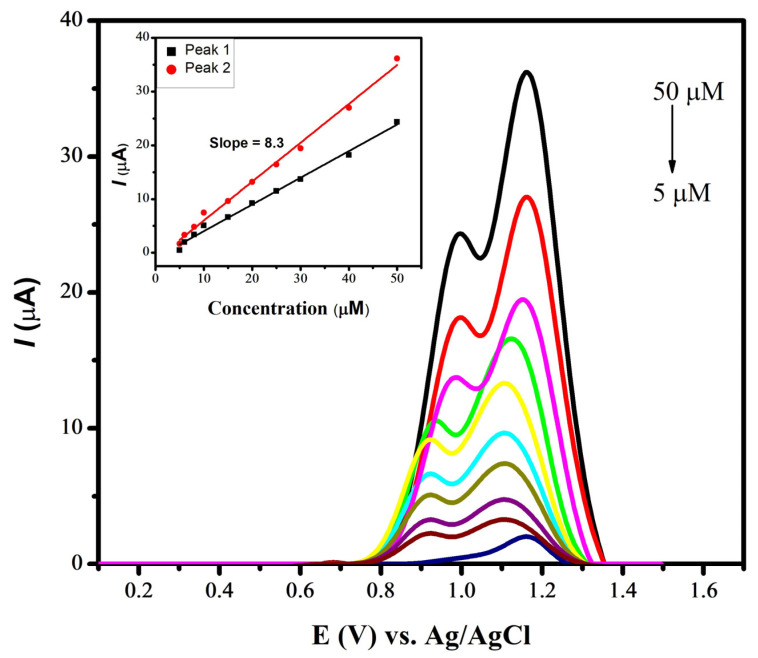
SWVs of various concentrations of naproxen using CdS/GCE in PBS of pH 7.0.

To assess the limit of detection (LOD) for naproxen utilizing a CdS-modified glassy carbon electrode, square-wave voltammetry was employed in a pH 7 phosphate-buffered solution. The process involved applying an accumulation potential of 0.3 V for 15 s, as illustrated in Figure 7. The peak current was found to linearly increase with the increase in the concentration of naproxen, ranging from 5 µM to 50 µM. The voltammograms of the blanks (supporting electrolyte solutions containing no naproxen) were recorded for reference. These measurements were instrumental in determining the standard deviation, allowing for a more precise evaluation of the LOD for naproxen under specific experimental conditions. The following equation was applied for the determination of the limit of detection, using the data obtained from the designed sensor:

LOD = 3 *σ*/m (2)

The designed sensor for the electrochemical detection of naproxen helped in achieving a LOD with a value of 1.43 nM. Similarly, a LOD with a value of 7.12 nM was evaluated for mobic with a zinc oxide-modified GCE under optimized conditions of a BRB of pH 4, a deposition time of 20 s and a deposition potential of −0.1 V. Table 1 illustrates the comparison of current work with the literature reported works.

## 4. Conclusions

Cadmium sulfide and zinc oxide nanoparticles were successfully synthesized and utilized as electrode modifiers that enhanced the sensitivity characteristics of a glassy carbon electrode for the sensing of naproxen and mobic. In comparison to an unmodified glassy carbon electrode, the oxidation signals of the NSAIDs intensified when using the designed sensing platforms due to the electrocatalytic and surface area enhancement roles of the cadmium sulfide and zinc oxide nanoparticles. The synthesized nanomaterials were immobilized individually on the electrode’s surface and its modification was ensured by square-wave voltammetry. The pH-dependent signals of the target NSAIDs, shifting in potential with the change in the pH of the medium, revealed proton-coupled electron transfer. The conditions were optimized to achieve the highest possible current response of the target analytes. The obtained results revealed the high sensitivity of the designed electrochemical platforms. The application horizon of the designed sensors can be extended to the investigation of the redox fate of NSAIDs in biological molecules and the monitoring of their metabolites in human serum and urine, as required for clinical diagnostics and for obtaining insights into the action mechanism of drugs.

## Figures and Tables

**Table 1:** Comparison of the performances of the designed CdS/GCE and ZnO/GCE sensors with reported sensing platforms.

Sr. No	Electrode Modifier	Technique	Supporting Electrolyte/pH	LOD of Naproxen	Ref.
1	Graphene oxide	DPV	PBS/7.2	6.47 µM	[24]
2	Reduced graphene oxide	CV	PBS/6.0	0.33 µM	[25]
3	MWCNTs/msCYP1A2-SPE	CV	PBS/7.4	16 µM	[26]
4	Ni-Co LDH/SPE	DPV	PBS/7.0	0.002 µM	[27]
5	Ni-Fe LDH/Au electrode	DPV	KCl/7.0	0.32 µM	[28]
6	ZnFe_2_O_4_/graphene oxide	DPV	PBS/7.0	0.222 nM	[29]
7	TsPro-Cs/rGO	DPV	PBS/7.0	0.4 μM	[30]
8	Poly l-serine/GCE	CV	PBS/5.0	0.69 μM	[31]
9	L-Cys/RGO/GCE	CV	PBS/7.0	0.35 µM	[32]
10	**CdS/GCE**	**SWV**	**PBS/7.0**	**1.43 nM**	**Current work**
Sr. No	Electrode modifier	Technique	Supporting Electrolyte/pH	LOD of mobic	Ref.
1	L-lysine/graphene oxide	DPV	BRB/pH 3	8.7 × 10^−7^ M	[33]
2	Carbon paste/polymer Nps-MWCNTs	DPV	PBS/pH 9.0	0.32 µM	[34]
3	Boron-doped diamond Electrode	DPV	BRB/pH 1–3	59 nM	[35]
4	Cystic acid/GCE	CV	BRB/pH 1.86	1.5 nM	[36]
5	Hg drop electrode	Polarography	Acetate Buffer/pH 4.88	57 nM	[37]
6	3.33% graphene/CPE	Adsorptive SWV	BRB/pH 2.0	2.59 nM	[38]
**7**	**ZnO/GCE**	**SWV**	**BRB/pH 4.0**	**7.21 nM**	**Current work**

## Data Availability

The data presented in this study are available on request from the corresponding author.
